# Supplementary material for: Role of Clinical Characteristics and Biomarkers at Admission to Predict One-Year Mortality in Elderly Patients with Pneumonia
Source: J Clin Med. 2021 Dec 25;11(1):105. doi: 10.3390/jcm11010105 (PMC8745347; doi:10.3390/jcm11010105)
Supplement: Supplementary file 1 [file jcm-11-00105-s001.zip › jcm-1504520-supplementary.pdf]

Supplementary Materials:

S1. PneumOldCT Study Group.

Complete membership of the PneumOldCT study group:

The leader of the group is Dr Virginie Prendki (principal investigator), virginie.prendki@hcuge.ch.

Other members: (in alphabetical order):

T Agoritsas, S Carballo, P Darbellay Farhoumand, C Marti, JL Reny, S Rosset-Zufferey, Jacques Serratrice, V. Soulier, J Stirnemann (co-investigator): Division of Internal Medicine, Department of Internal Medicine Specialties, Geneva University Hospitals, Switzerland

C. Combescure: Clinical Research Center, Geneva University and Hospitals Geneva University, Switzerland

N Garin: Department of General Internal Medicine, Riviera Chablais Hospitals, Switzerland

F Herrmann, V Lachat, MP Meynet, X Roux, C Serratrice, Department of Rehabilitation and Geriatrics, Geneva University Hospitals, Switzerland

X Montet, M Scheffler, Department of Radiology, Geneva University Hospitals and University of Geneva, Switzerland

B Huttner, L Kaiser. Division of Infectious Diseases, Department of Internal Medicine Specialties, Medical Faculty, Geneva University and Hospitals Geneva University, Switzerland

JP Janssens, Division of Pulmonology, Department of Internal Medicine Specialties, Geneva University Hospitals and University of Geneva, Switzerland
